# The Bacterial Symbionts of Closely Related Hydrothermal Vent Snails With Distinct Geochemical Habitats Show Broad Similarity in Chemoautotrophic Gene Content

**DOI:** 10.3389/fmicb.2019.01818

**Published:** 2019-08-14

**Authors:** Roxanne A. Beinart, Chengwei Luo, Konstantinos T. Konstantinidis, Frank J. Stewart, Peter R. Girguis

**Affiliations:** ^1^Graduate School of Oceanography, University of Rhode Island, Narragansett, RI, United States; ^2^School of Civil and Environmental Engineering, Georgia Institute of Technology, Atlanta, GA, United States; ^3^School of Biological Sciences, Georgia Institute of Technology, Atlanta, GA, United States; ^4^Department of Organismic and Evolutionary Biology, Harvard University, Cambridge, MA, United States

**Keywords:** symbiosis, chemosynthesis, genomics, *Campylobacteria*, *Gammaproteobacteria*, gastropod, *Alviniconcha*, *Ifremeria*

## Abstract

Symbiosis has evolved between a diversity of invertebrate taxa and chemosynthetic bacterial lineages. At the broadest level, these symbioses share primary function: the bacterial symbionts use the energy harnessed from the oxidation of reduced chemicals to power the fixation of inorganic carbon and/or other nutrients, providing the bulk of host nutrition. However, it is unclear to what extent the ecological niche of the host species is influenced by differences in symbiont traits, particularly those involved in chemoautotrophic function and interaction with the geochemical environment. Hydrothermal vents in the Lau Basin (Tonga) are home to four morphologically and physiologically similar snail species from the sister genera *Alviniconcha* and *Ifremeria*. Here, we assembled nearly complete genomes from their symbionts to determine whether differences in chemoautotrophic capacity exist among these symbionts that could explain the observed distribution of these snail species into distinct geochemical habitats. Phylogenomic analyses confirmed that the symbionts have evolved from four distinct lineages in the classes γ*-proteobacteria* or *Campylobacteria*. The genomes differed with respect to genes related to motility, adhesion, secretion, and amino acid uptake or excretion, though were quite similar in chemoautotrophic function, with all four containing genes for carbon fixation, sulfur and hydrogen oxidation, and oxygen and nitrate respiration. This indicates that differences in the presence or absence of symbiont chemoautotrophic functions does not likely explain the observed geochemical habitat partitioning. Rather, differences in gene expression and regulation, biochemical differences among these chemoautotrophic pathways, and/or differences in host physiology could all influence the observed patterns of habitat partitioning.

## Introduction

Symbiosis with chemosynthetic bacteria has independently evolved in many hydrothermal vent, cold seep, and shallow-water invertebrate taxa. Broadly speaking, the animal hosts rely on the fixed carbon produced by their obligate symbionts for the majority of their nutrition (Dubilier et al., 2008). The bacterial partners of such associations are phylogenetically diverse, representing multiple bacterial lineages across two phyla. Nevertheless, with respect to energy metabolism, they perform similar metabolic functions: these symbionts use reduced chemicals like hydrogen sulfide as electron donors, respire oxygen and/or nitrate, and fix inorganic carbon into organic carbon (Dubilier et al., 2008; Childress and Girguis, 2011).

Chemoautotrophic symbionts can, at times, exhibit marked differences in gene content, which can subsequently confer differences in metabolism that may have ecological implications for their host species. For example, a comparison of the genomic content among some of the γ*-proteobacterial* symbionts of vent animals has shown that they can differ in a diversity of genes and gene networks, from energy metabolism to nitrogen acquisition (Kleiner et al., 2012). While it is reasonable to assert that genomic content that confers symbiont functional traits could affect the distribution of host species into distinct physicochemical habitats, it is still unknown whether this occurs. Interpretation of the similarities and differences in the genomes of chemosynthetic symbionts has been confounded by the fact that the comparisons to date have been made among symbionts from highly divergent host taxa and/or from different geographical locations with distinct environmental characteristics and biogeographic histories. Thus, it is difficult to untangle whether observed differences in symbiont gene content are driven by co-evolution with physiologically and ecologically distinct host taxa, or adaptation to dissimilar habitats. Comparisons among the symbionts of closely related host species can help resolve whether differences in symbiont genomic traits impact their distribution into distinct geochemical habitats.

The hydrothermal vent snail genera *Alviniconcha* and *Ifremeria* provide a unique opportunity to examine gene content differences among the symbionts of closely related and regionally sympatric host species that occupy distinct geochemical habitats. *Alviniconcha* and *Ifremeria* are sister genera of provannid gastropod molluscs (snails) that dominate hydrothermal vent communities in the southwestern Pacific (Desbruyères et al., 1994). At vents along the Eastern Lau Spreading Center (Tonga), three species of *Alviniconcha* (*A. boucheti*, *A. kojimai*, and *A. strummeri*), co-occur with each other and with *Ifremeria nautilei* (Beinart et al., 2012; Johnson et al., 2015). These snail species associate with a total of four distinct phylotypes from the phylum *Proteobacteria* (class γ*-proteobacteria*) and one from the phylum *Campylobacterota* (*class Campylobacteria*), which are all hosted as intracellular gill endosymbionts (Suzuki et al., 2005a,b,2006a,b; Urakawa et al., 2005; Beinart et al., 2012). The highly reduced gut and carbon stable isotopic composition of host tissue indicates that these symbionts provide nutrition for their hosts through chemoautotrophy (Waren and Bouchet, 1993; Suzuki et al., 2005a,b,2006a,b; Beinart et al., 2012). Since they represent different bacterial lineages and do not show a pattern of co-divergence with host species (Suzuki et al., 2006a; Beinart et al., 2012), the symbionts are thought to be acquired horizontally from the environment. However, there is strong specificity between each snail species and its symbiont phylotypes(s): surveys of hundreds of individuals across a range of habitats have demonstrated that each snail species associates with only one or two of the symbiont phylotypes, with individual snails usually dominated by just one symbiont phylotype at a time (Table 1; Beinart et al., 2012; Seston et al., 2016).

**TABLE 1:** Specificity between host species and possible symbiont phylotypes, with symbiont taxonomy to the level of class.

**Host species**	**Possible dominant phylotypes (Taxonomic class)**	**Dominant phylotype in sequenced individual**	**Vent field**	**Lat**	**Lon**	**Depth (m)**
*A. kojimai*	ε (*Campylobacteria*)	γ-1	ABE	2045.80*S*	17611.48*W*	2146
*A. strummeri*	γ-1 (*Gammaproteobacteria*)	γ-Lau	Tu′i Malila	2159.37*S*	17634.11*W*	1871
*A. boucheti*	γ-1 or γ-Lau (*Gammaproteobacteria*)	ε	Kilo Moana	203.23*S*	1768.01*W*	2614
*I. nautilei*	Ifr1 (*Gammaproteobacteria*)	Ifr1	ABE	2045.80*S*	17611.48*W*	2146

*Additionally, the dominant symbiont phylotype, as well as sampling location and depth for the sequenced snail specimens from each species.*

As such, it is plausible that differences in gene content among these symbionts could influence the geochemical niche of the host snails at the Eastern Lau Spreading Center. Indeed, each snail species is restricted to associations with only one or two of the symbiont phylotypes, so differences in symbiont physiological traits could partition host species into distinct habitats based on these traits. The four snail species have similar anatomy in terms of overall body size, as well as gill and circulatory structure (Waren and Bouchet, 1993; Johnson et al., 2015). Despite these shared features, *Alviniconcha* and *I. nautilei* occupy distinct geochemical habitats (Waite et al., 2008; Podowski et al., 2009, 2010; Beinart et al., 2012), and this segregation is thought to primarily be driven by the distribution of reducing compounds that could be used by their particular symbionts for chemoautotrophy (Henry et al., 2008; Beinart et al., 2012, 2015; Sanders et al., 2013). Within a single vent field, *Alviniconcha* and *I. nautilei* show consistent patterns of zonation, with *Alviniconcha* species found nearest to vent orifices and *I. nautilei* on the edges of *Alviniconcha* patches (Podowski et al., 2009, 2010). Thus, compared to *I. nautilei*, *Alviniconcha* species occur where concentrations of vent-derived reductants and temperatures are higher, and oxygen and thiosulfate concentrations are lower (Waite et al., 2008; Podowski et al., 2009, 2010). However, at a regional scale, the dominant *Alvinconcha* species at each vent field varies according to the particular geochemistry of that site (Beinart et al., 2012). Specifically, the *Alviniconcha* species (*A. boucheti*) hosting the *Campylobacterial* symbiont dominates at vent fields with higher concentrations of hydrogen and hydrogen sulfide, whereas the *Alviniconcha* species (*A. kojimai* and *A. strummeri*) hosting γ*-proteobacterial* symbiont phylotypes dominate at vents with lower concentrations of these two reductants (Beinart et al., 2012). These vent fields are separated by 10 s to 100 s of kilometers, but are part of one biogeographic region with no known barriers to dispersal among sites (Speer and Thurnherr, 2012; Mitarai et al., 2016), suggesting that the distribution of *Alviniconcha* species could be tied to how their specific symbionts interact with the varying concentrations of chemical reductants at each site. Physiological experiments have directly demonstrated that some of these species can oxidize hydrogen sulfide and thiosulfate to support high rates of carbon fixation (Henry et al., 2008; Beinart et al., 2015). In addition, studies of gene transcription *in situ* and in laboratory experiments have demonstrated differences in expression of genes for sulfur oxidation, hydrogen oxidation, and both assimilatory and dissimilatory nitrate reduction (Sanders et al., 2013; Seston et al., 2016). These studies provide an understanding of metabolic pathway use and rates under conditions at the time of sampling and treatment, but do not allow for a comparison of the full metabolic and physiological potential of each symbiont.

To investigate whether variation in chemoautotrophic capacity (i.e., the genomic content of each specific symbiont) confers differences in energy and carbon metabolism that play a role in the distribution of the host species, we assembled the genomes of the dominant four symbiont phylotypes associated with *Alviniconcha* species and *I. nautilei* from the Eastern Lau Spreading Center (Tonga). We specifically concentrate on comparisons of the genes for chemoautotrophic metabolism of these symbionts, since the host species segregate into distinct niches that differ primarily in the chemicals used by the symbionts for chemoautotrophy (hydrogen, sulfur) (Waite et al., 2008; Podowski et al., 2009, 2010; Beinart et al., 2012), suggesting that their symbionts could differ in their capacity to use these chemicals. Subsequent phylogenomic and comparative analyses with these nearly complete genomes demonstrated that the symbionts, although representing multiple lineages, exhibit broad similarity in the representation of genes associated with chemoautotrophic energy generation and biosynthesis. These data underscore that gene content alone is not robust as a causal factor for the observed habitat partitioning among these symbioses. Rather, these data raise alternative hypotheses about the factors controlling habitat partitioning, for example the extent to which differential gene expression and regulation, biochemical differences in the particular pathways employed by the symbionts, and the possibility of uncharacterized differences in host or symbiont physiology, play a role in governing the distribution of *Alviniconcha* species and *I. nautilei*.

## Materials and Methods

### Sample Collection

Snails were collected from the ABE, Tu’i Malila, and Kilo Moana vent fields at the Eastern Lau Spreading Center (Table 1) by the remotely operated vehicle *JASON II* during the R/V *Thomas G. Thompson* expedition TM-235 in 2009. *Alviniconcha* were recovered in insulated containers and, once on board, were briefly kept in 4°C seawater until dissection of symbiont-containing gill tissue. Gill tissues were stored at −80°C until DNA extraction. DNA was extracted from 25 mg subsamples of frozen gill tissue using the AutoGenprep 965 automatic extraction system with the AutoGenprep 965/960 Tissue DNA Extraction kit (AutoGen, Inc.), as previously described (Beinart et al., 2012). The *I. nautilei* individual used for sequencing was part of a high-pressure physiological experiment (see Beinart et al., 2015). A the conclusion of the experiment, symbiont-containing gill tissue was dissected, homogenized in Trizol^TM^ (Thermo Fisher Scientific, Inc.) preservative, and stored at −80°C until DNA extraction as described in Seston et al. (2016).

### Sequencing, Genomic Bin Assembly, and Annotation

Gill DNA consists of both host and symbiont genomic material. Thus, the resulting metagenomic sequences are a metagenomic mixture of both host and symbiont genomes. Two separate approaches were used to assemble the symbiont genomes from these metagenomes. For the symbionts of *Alviniconcha*, a novel method was developed to separate symbiont reads from host reads prior to assembly (described herein). For the *I. nautilei* symbionts, all reads were assembled, and then binning of contigs based on an Expectation-Maximization algorithm was used to separate symbiont contigs from each other and the host genome (Wu et al., 2014).

Three *Alviniconcha* and one *I. nautilei* individuals were selected for sequencing (Table 1). Each *Alviniconcha* individual was dominated by only one of the three phylotypes of bacterial symbiont, as previously assessed via symbiont-specific quantitative PCR assays (see Beinart et al., 2012). The *I. nautilei* individual (Table 1) had been previously shown, via 16S rRNA gene amplicon sequencing, to be dominated by one major symbiont phylotype from the order *Chromatiales* (Ifr1), with a very small sub-population of a second phylotype from the family *Methylococcaceae* in some individuals (Ifr2) (Seston et al., 2016). Symbiont 16S rRNA gene sequence similarity is >98% among host individuals sharing the same symbiont phylotype (Beinart et al., 2012, 2015; Seston et al., 2016). This low level of intra-phylotype diversity is at approximately the threshold typically used to differentiate bacterial species (Konstantinidis et al., 2017). Therefore, each sequenced individual (host) in our study acts as a representative for its phylotype, with each phylotype constituting a sub-species-level population. In addition, the *Alviniconcha* individuals were genotyped via amplification and sequencing of the host mitochondrial CO1 gene (to date *Alviniconcha* species cannot be distinguished morphologically, see (Beinart et al., 2012; Johnson et al., 2015; Table 1).

Gill DNA from the three *Alviniconcha* individuals was used for Illumina HiSeq sequencing (read length: 2 × 150 bp). Reads were trimmed using the protocol described in Luo et al. (2011), followed by a *K*-mer-based algorithm to separate the *Alviniconcha* reads and the bacterial reads. The algorithm leverages the fact that the *Alviniconcha* snail genome is significantly larger than the bacterial genome, and hence, random reads generation would result in a bivariate normal distribution in read coverage. Such a bivariate normal distribution would be reflected in *K-*mer frequency, which can be used as described below as a method for read separation:

Assuming the *Alviniconcha* genome *S* size is *s*, and the symbiont bacterial genome Q size is *q*, for a given K-mer, *k*_*i*_, its occurrence in the *Alviniconcha* genome is *N*_*s*_(*k*_*i*_) and its occurrence in the bacterial genome is *N_*g*__1_*(*k*_*i*_). Also, assuming that there are *n* reads of *l* –bp long each in the sample, and denoting the *Alviniconcha* reads’ fraction, *R*, then the number of *Alviniconcha* base pairs in reads is *nlR*, and the number of bacterial base pairs in reads is *nl*(*1-R*). Assuming DNA sequencing covers the genomes uniformly, for a given *K*-mer, *k*_*i*_, the expected observance, *N*_*m*_(*k*_*i*_), is:

$$N_{m}\,\left(k_{i}\right)=N_{s}\,\left(k_{i}\right)\,n\,l\,R/s+N_{Q}\,\left(k_{i}\right)\,n\,l\,\left(1-R\right)/q$$

We denote the *K*-mers a read contains as *K* = *{k_1_*, *k*_2_, …, *k_*m*_}* with corresponding occurrences *O* = *{o_1_*, *o*_2_, …, *o_*m*_}*. We can transform occurrences into ranks, denoted as *R* = *{r_1_*, *r*_2_, …, *r_*m*_}.* With these, the likelihood that a paired-end read, *r*, is from the symbiotic bacterial genome is:

$$\left(r\in q|O\right)=\frac{P\left(O|r\in q\right)P\left(r\in q\right)}{P\left(O|r\in q\right)P\left(r\in q\right)+P\left(O|r\in s\right)P\left(r\in s\right)}$$

*P*(*r* ∈ *q*) and *P*(*r* ∈ *q*) represent the probability of a read originating from the bacterial genome and from the *Alviniconcha* genome, respectively, and they can be initialized by using the average genome sizes of the closest relative available. In our practice, we have found that since the genome sizes are significantly different, the accuracy of the genome size estimation has little effect on the end results.

*P*(*O*|*r* ∈ *q*) is the conditional probability of observing such *K-mer* occurrences as in *O* if a read was originated from the symbiotic bacterial genome *q*, and it was calculated as:

$$\left(O|r\in q\right)=\sum_{j=1}^{m}P\left(o_{j}|r\in q\right)$$

where *P*(*o*_*j*_|*r* ∈ *q*) can be approximated using the discrete ranks as:

$$P\left(o_{j}|r\in q\right)=\frac{\left|\left\{k:k\geq k_{j},k\in K\right\}\right|}{\left|\left\{k:k\geq 1,k\in K\right\}\right|}$$

where *K* is the set of all ranks of K-mers in the metagenome.

As such, for each read, we counted the *K-*mer occurrences using Jellyfish (Marçais and Kingsford, 2011), and calculated the probability of it originating from *Alviniconcha.* A K-means (*K* = 2) clustering was performed on to separate the reads. The Velvet assembler (Zerbino and Birney, 2008) was then used with *K* = 41 and default settings to assemble the reads into a cluster identified as the symbiotic bacterium.

Gill DNA from *I. nautilei* was sequenced on an Illumina MiSeq (2 × 300 bp). Resulting sequences were quality filtered and trimmed with TrimGalore!^1^ using a read quality cutoff of Q25, read length cutoff of 100 and Nextera flag for adapter detection. Approximately 21 million read pairs were assembled using IDBA-UD with default parameters (Peng et al., 2012). Using MaxBin v1.4.5 (Wu et al., 2014), two *I. nautilei* symbiont genome bins were recovered from the 294 Mb assembly that included both host and symbiont contigs.

In all cases, only contigs >500 bp were used for downstream analyses of the putative symbiont genome bins. Symbiont genome assembly characteristics, completeness, contamination, quality, and taxonomy assignment were assessed via the Microbial Genomes Atlas (MiGA) webserver (Rodriguez-R et al., 2018). Gene prediction and annotation were done with RASTtk (Brettin et al., 2015) using the default workflow. Sequences annotated as hydrogenases were further classified with HydDB (Søndergaard et al., 2016).

### Phylogenomic Analysis

All *Chromatiales* (NCBI Tax. ID: 135613), *Sulfurimonas* (NCBI Tax. ID 202746), and sulfur-oxidizing symbiont (NCBI Tax. ID: 32036) genomes from NCBI were placed into a bacterial phylogeny with the *Alviniconcha* and *I. nautilei* symbiont genomes using a 6,989 position amino acid alignment of 43 concatenated phylogenetically informative marker genes with PPlacer (Matsen et al., 2010) in CheckM (Parks et al., 2015). A subset of the genomes most closely related to each of the *Alviniconcha* and *I. nautilei* symbiont genomes were selected for further phylogenetic analysis, and the marker gene alignment of each subset was exported from CheckM (Parks et al., 2015). The best model for amino acid evolution was determined for each alignment using ModelGenerator v0.851 (Keane et al., 2006): LG + G + F for the *Alviniconcha* ε and γ-1 alignments, and LG + I + G + F for the alignment containing the two other symbionts (γ-Lau and Ifr1). Maximum likelihood phylogenies were constructed with RAxML v.8.2.10 (Stamatakis, 2014) via the CIPRES web server (Miller et al., 2010). Clade support was estimated using bootstrapping halted automatically by RAxML. Average amino acid identities (AAI) were calculated for each symbiont and its closest relative using the AAI tool that is part of the enveomics collection (Rodriguez-R and Konstantinidis, 2016).

## Results and Discussion

### Genome Assemblies

A single symbiont genome, representing the previously identified dominant symbiont, was assembled from each *Alviniconcha* individual, while two symbiont genomes were assembled from the *I. nautilei* individual (herein, called Ifr1 and Ifr2). Each symbiont genome contained a single-copy 16S rRNA gene that had 99% identity with the published 16S rRNA genes for the symbionts of *Alviniconcha* and *I. nautilei* in the Lau Basin (Beinart et al., 2012; Seston et al., 2016). The 16S rRNA genes from each genome also matched the dominant symbiont phylotype expected for each individual based on previous assessment of the symbionts associated with each snail by qPCR or 16S rRNA gene amplicon sequencing (Beinart et al., 2012; Seston et al., 2016). The *Alviniconcha* symbionts had previously been called the ε, γ-Lau, and γ-1 symbiont phylotypes (Beinart et al., 2012; Johnson et al., 2015). Taxonomy assignment based on marker gene sequences was possible to the level of family for the *A*. ε symbiont (*Helicobacteraceae*) and the Ifr2 symbiont (*Methylococcaceae*). The *A.* γ-Lau, Ifr1, and *A*. γ-1 symbiont were classified to the level of the class γ-*proteobacteria* (Table 2). Because the Ifr2 symbiont genome bin, which represents the minority symbiont in *I. nautilei* (Seston et al., 2016), was large (∼8 Mbp), highly fragmented, had a lower estimated completion (62%), relatively high level of contamination (6%), and intermediate quality (31%) (Table 2), it was not included in further analysis. In contrast, the other four symbiont genomes were near complete (87–96%), with low estimated contamination (<4%), high to excellent quality (69–90%), sizes from 2.0 to 3.7 Mb, and GC content from 33 to 59% (Table 2).

**TABLE 2:** Characteristics of symbiont genomes including the percent completeness (Comp.) and contamination (Cont.), the total size, average GC content, number of contigs, N50 of the contigs, and taxonomy assigned to lowest possible rank.

**Genome**	**Comp. (%)**	**Cont. (%)**	**Qual. (%)**	**Assembly Size (Mb)**	**Avg. GC**	**No. scaffolds**	**N50 scaffolds (bp)**	**Taxonomy (*p*-value)**
Ifr1	86.5	3.6	68.5	3.05	0.56	971	4,603	γ*-proteobacteria* (0)
Ifr2	62.2	6.3	30.7	7.98	0.48	9,656	825	*Methylococcaceae* (0.022)
*A.* γ-1	95.5	1.8	86.5	3.67	0.43	1,391	4,956	γ*-proteobacteria* (0)/*Chromatiales* (0.346)
*A.*γ-Lau	94.6	0.9	90.1	2.10	0.59	140	101,222	γ*-proteobacteria* (0)
*A.* ε	92.8	1.8	83.8	1.96	0.33	283	22,517	*Helicobacteraceae* (0.00086)/*Sulfurimonas* (0.297)

### Phylogenomic Analysis

**FIGURE 1:**
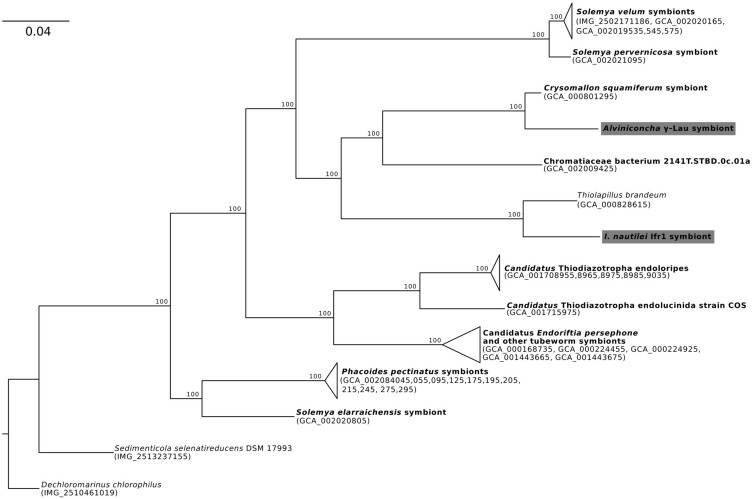
Maximum-likelihood phylogenomic analysis of a 6,989 position concatenated protein alignment of 43 marker genes from the *Alviniconcha* γ-Lau symbiont, *I. nautilei* Ifr1 symbiont, and relatives using the LG + I + G + F model of protein evolution. Symbiont genomes from this study are highlighted in gray, and genomes derived from host-associated symbionts are shown in bold. Percent bootstrap support for each clade shown. The scale bar represents the mean number of nucleotide substitutions per site.

**FIGURE 2:**
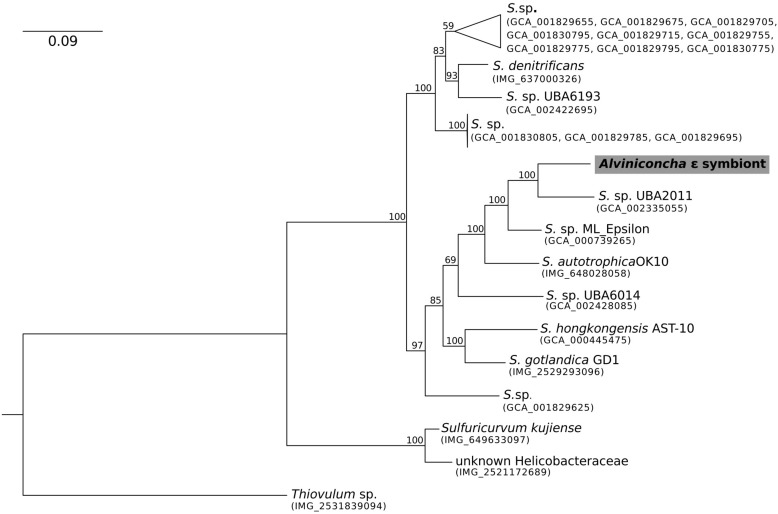
Maximum-likelihood phylogenomic analysis of a 6,989 position concatenated protein alignment of 43 marker genes from the *Alviniconcha* ε symbiont and its *Sulfurimonas* relatives using the LG + G + F model of protein evolution. The symbiont genome from this study is highlighted in gray. Percent bootstrap support for each clade shown. The scale bar represents the mean number of nucleotide substitutions per site.

**FIGURE 3:**
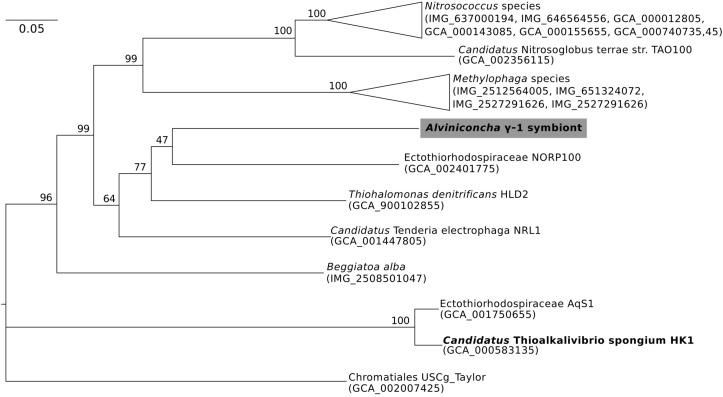
Maximum-likelihood phylogenomic analysis of a 6,989 position concatenated protein alignment of 43 marker genes from *Alviniconcha* γ-1 and relatives using the LG + G + F model of protein evolution. The symbiont genome from this study is highlighted in gray, and genomes derived from host-associated symbionts are shown in bold. Percent bootstrap support for each clade shown. The scale bar represents the mean number of nucleotide substitutions per site.

Phylogenomic analyses of the four symbiont genomes based on 43 marker genes showed that each was affiliated with a different clade of γ*-proteobacteria* or *Campylobacteria* (Figures 1–3), confirming previous 16S rRNA gene phylogenies and indicating that the *Alviniconcha* and *I. nautilei* symbionts were acquired separately by each host species (Urakawa et al., 2005; Suzuki et al., 2005a, b,2006a, b; Beinart et al., 2012). The *A*. γ-Lau and Ifr1 symbionts clustered in a clade that is entirely comprised of genomes assembled from host-associated, chemoautotrophic symbionts, with the exception of that from *Thiolapillus brandeum* (NCBI Assembly: GCA_000828615), a free-living close relative of the Ifr1 symbiont that was isolated from a western Pacific hydrothermal vent (Nunoura et al., 2014; Figure 1). *T. brandeum* has 97% identity with the Ifr1 16S rRNA gene and a two-way average amino acid identity (AAI) of 76% (1998 proteins). *A*. γ-Lau is very closely related to the symbiont of *Chrysomallon squamiferum*, a snail from hydrothermal vents in the Indian Ocean (Nakagawa et al., 2014), showing 97% 16S rRNA gene identity, and 88% two-way AAI (1547 proteins). These 16S rRNA gene identities and AAIs represent values that are typically seen between bacterial species from the same genus (Rodriguez-R and Konstantinidis, 2014).

In contrast, the closest relatives of the other two *Alviniconcha* symbiont genomes were from environmental samples, not known to be associated with a host, and were much more divergent from the symbionts (Figures 2, 3). The *A*. ε symbiont clustered within a clade of bacteria in the genus *Sulfurimonas* and was most closely related to a *Sulfurimonas* metagenome-assembled genome (MAG) from a hydrothermal vent plume in the Caribbean (NCBI Assembly: GCA_002335055) (Figure 2). This *Sulfurimonas* MAG does not contain a 16S rRNA gene, but showed 36% two-way AAI (1139 proteins) with the *A*. ε symbiont genome. The *A*. γ-1 symbiont clustered within a larger clade that included both free-living *Methylophaga* and *Nitrosococcus* species, but its closest relative was a MAG from an oceanic subsurface aquifer (NCBI Assembly: GCA_002401775) (Figure 3). This MAG does not contain a 16S rRNA gene, but showed 50% two-way AAI (1552 proteins) with the *A*. γ-1 symbiont.

### Overall Gene Content

**FIGURE 4:**
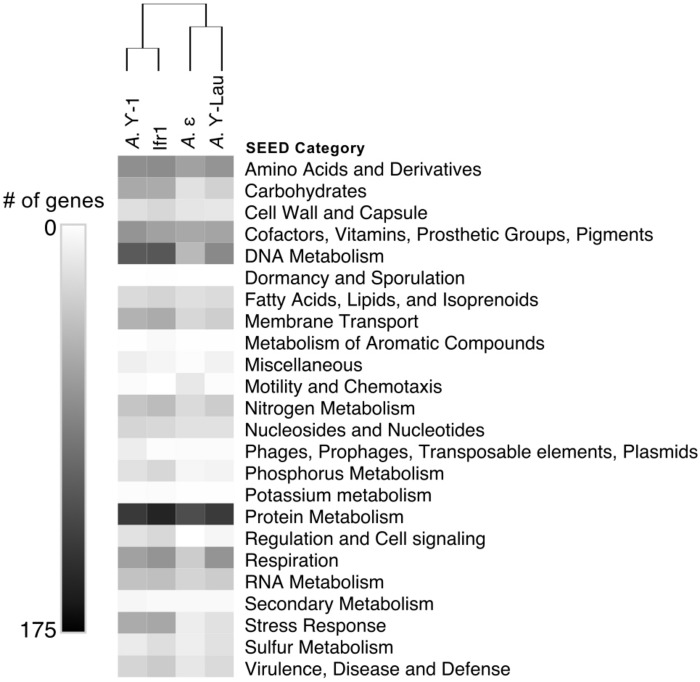
Heatmap showing the number of genes annotated to each SEED category within each symbiont genome. Hierarchical clustering based on Euclidean distance with average linkage.

**FIGURE 5:**
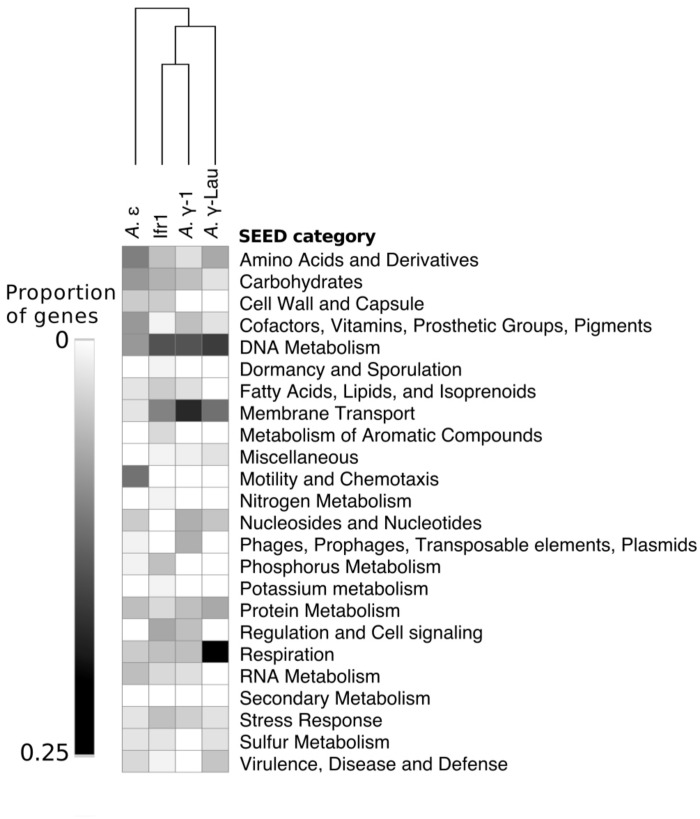
Heatmap showing the proportion of unique genes (i.e., those not present in any other genome) annotated to each SEED category from each symbiont genome. Hierarchical clustering based on Euclidean distance with average linkage.

Between 2072 and 4214 gene coding sequences were found in each symbiont genome assembly (Supplementary Tables S1, S2). Each assembly contained a single-copy 23S rRNA gene in addition to the 16S rRNA gene (reported above). However, the 5S rRNA gene was present in the Ifr1 and *A.* γ-Lau symbiont assemblies only. Of the protein-coding sequences, approximately half in each genome were annotated as hypothetical proteins (Supplementary Table S1). The overall composition of functional genes (evaluated at the level of SEED category) was relatively similar among genomes – in all genomes, the most abundant gene categories were “Protein Metabolism” and “Amino Acids and Derivatives” (Figure 4). The proportion of genes annotated to the “DNA Metabolism” category was high in all three γ*-proteobacterial* symbiont genome assemblies, but lower in the *A*. ε genome assembly (Figure 4). The four symbiont genomes shared 586 core orthologs (Supplementary Tables S2, S3), which represent 36–50% of the genes in each genome. Of the 59% of the core orthologs that could be placed into SEED categories, the most abundant were placed in “Protein metabolism,” “Amino acids and derivatives,” and “Cofactors, Vitamins, Prosthetic Groups, Pigments;” altogether, these three categories represented about a third of the core orthologs (Supplementary Table S3). Each genome also encoded between 129 and 345 genes with unique annotations not found in any of the other three genomes, representing 10–30% of the total annotations in each genome (Supplementary Table S2). In all four genomes, most of the unique genes could not be put into SEED categories. Of the unique genes that could be classified by SEED, most of those from the γ*-proteobacterial* symbiont genomes were in either the “DNA metabolism” or “Membrane transport” categories (Figure 5), whereas those from the *A*. ε genome were primarily in the “Motility and chemotaxis” and “Amino acids and derivatives” categories (Figure 5).

### Sulfur Oxidation

All of the snail symbiont genomes revealed pathways for the oxidation of both hydrogen sulfide and thiosulfate. As suggested in a previous analysis of symbiont gene expression (Sanders et al., 2013; Seston et al., 2016), all four symbiont genomes encoded the thiosulfate-oxidizing Sox multienzyme complex (*soxABXYZ*), with only the *Alviniconcha* ε symbiont encoding the genes *soxCD*. The lack of *soxCD* in the γ*-proteobacterial* symbionts is typical of this taxonomic class (Gregersen et al., 2011), and usually results in only partial oxidation of sulfur to elemental sulfur in the periplasm. The γ*-proteobacterial* symbiont genomes encoded genes for the complete oxidation of this elemental sulfur through the reverse dissimilatory sulfate reduction pathway (*rdsrABMKOP*), APS-reductase (*aprAB*), and sulfate adenylyltransferase (*sat*).

In addition, all four symbiont genomes contained genes for the oxidation of hydrogen sulfide to elemental sulfur in the periplasm. All of the symbiont genomes contained a type VI sulfide:quinone oxidoreductase (*sqrF*), which has been shown to be important for growth at high sulfide concentrations (>4 mM) in a photosynthetic sulfur-oxidizer (Gregersen et al., 2011). Each genome also contained an additional type of sulfide:quinone oxidoreductase: the γ*-proteobacterial* symbionts each additionally encoded a type I (*sqrA*), while the *A*. ε symbiont encoded a type IV (*sqrD*). Finally, the Ifr1 symbiont and the *A*. γ-1 symbiont genome each additionally had a gene for a flavocytochrome c:sulfide dehydrogenase (*fcc*) which is also thought to oxidize sulfide to elemental sulfur or polysulfides in the periplasm (Chen et al., 1994). These particular sets of *sqr* and *fcc* genes are common in the symbionts’ respective taxonomic groups (Gregersen et al., 2011; Han and Perner, 2015), and may be useful for dealing with different concentrations of available sulfide (Han and Perner, 2016).

All four snail symbiont genomes encoded at least one gene for a sulfate permease or transporter, which could be used for the excretion of sulfate (the end-product of sulfur oxidation) or the uptake of thiosulfate (Aguilar-Barajas et al., 2011). Hydrogen sulfide can freely diffuse across membranes (Mathai et al., 2009), while hydrosulfide ions (HS-) must move through a membrane channel (Czyzewski and Wang, 2012). No genes for a hydrosulfide ion channel, which is related to channels for nitrate and formate (Czyzewski and Wang, 2012), were detected in any of the snail symbiont genomes.

### Hydrogen Oxidation

Each of the symbiont genomes encoded genes for two types of hydrogenases, a respiratory version that enables the use of hydrogen as an electron donor, and regulatory version that allows for sensing and response to hydrogen concentrations. The *A*. ε symbiont genome contained a group 1b quinone-reactive NiFe hydrogenase (*hydABC*), which is a prototypical hydrogenase that is used for anaerobic respiration, for example with nitrate (Søndergaard et al., 2016). Each γ*-proteobacterial* symbiont genome contained a group 1d oxygen-tolerant NiFe hydrogenase (*hyaABC*) that typically pairs hydrogen oxidation with the respiration of oxygen or with oxygen-tolerant anaerobic respiration (Søndergaard et al., 2016). In addition to its respiratory hydrogenase, the *A*. ε symbiont genome contained genes for a NiFe hydrogenase (*hyaAB*) that is classified within the group 2d Aquificae-type hydrogenases, which currently have unknown function but are thought to either be regulatory or provide reducing power for carbon fixation (Søndergaard et al., 2016). The γ*-proteobacterial* symbiont genomes also each included a group 2b NiFe sensory hydrogenase (*hoxBC/hupUV*) that can sense hydrogen and regulate the expression of other hydrogenases (Peters et al., 2015). Based on previously observed variation in symbiont hydrogenase gene expression (Sanders et al., 2013) and the predominance of *Alviniconcha* species hosting the *A*. ε symbiont at vent fields with higher hydrogen concentrations (Beinart et al., 2012), it has been hypothesized that *Alviniconcha* symbionts have differing capacities for the use of hydrogen as an energy source. Notably, the gene content observed here suggests that all of these snail symbionts have the ability to oxidize hydrogen, though it remains to be tested whether hydrogen can provide energy for autotrophy and, if so, how hydrogen use varies depending on environmental conditions.

### Respiration

All of the snail symbiont genomes encoded the genes for aerobic respiration. All snail symbiont genomes contained genes for NADH-ubiquinone oxidoreductase (subunits ABCDEFGHIJKLMN, though subunit F is missing from the *A*. ε symbiont genome). The Ifr1, *A*. ε, and *A*. γ-1 symbiont genomes each had some of the genes for a succinate dehydrogenase. All of the symbiont genomes had all necessary genes for cytochrome bc1-type ubiquinol oxidoreductase. The Ifr1 and the *A*. γ*-proteobacterial* symbionts all had five genes for different types of cytochrome c, while the *A*. ε symbiont had only two cytochrome c genes. All had genes for the cytochrome *cbb*_3_-type oxidase (*ccoNOPQ*, though *ccoQ* was not found in the *A*. γ-1 symbiont genome). The Ifr1 symbiont genome also encoded subunits I and II for a cytochrome *bd*-type ubiquinol oxidase, which has a high affinity for oxygen (D’Mello et al., 1996). The presence of a high affinity cytochrome in the Ifr1 symbiont is unexpected, given that *I. nautilei* typically occupies habitats with relatively high oxygen concentrations (Podowski et al., 2009, 2010). The presence of this gene may indicate that the symbionts of I. *nautilei* experience relatively low intracellular oxygen concentrations, despite the environmental conditions.

The four symbiont genomes all also encoded genes for anaerobic respiration. For the symbionts of oxygen-respiring animals, the ability to utilize an electron acceptor other than oxygen may be useful as a way to avoid competing with host respiration. Additionally, hydrothermal vent habitats have the potential to be low in oxygen, which could necessitate the use of anaerobic respiration by the symbionts, either during association with a host or a free-living, environmental stage. All symbiont genomes encoded genes for a periplasmic nitrate reductase (*napABGH*), as well as for the complete denitrification pathway to nitrogen gas: nitric-oxide forming nitrite reductase (*nirKN*), nitric-oxide reductase (*norBC*), and nitrous-oxide reductase (*nosZ*). This suggests that all four symbionts can respire nitrate. In addition, the γ*-proteobacterial* symbiont genomes all contained genes for an anaerobic dimethyl sulfoxide (DMSO) reductase (*dmsABC*), which would allow them to respire dimethyl sulfoxide. Dimethyl sulfide (DMS) and other organic sulfur compounds are present around hydrothermal vents (Rogers and Schulte, 2012), and it is possible that DMSO is present in habitats occupied by *Alviniconcha* and *I. nautilei*, and could be used for anaerobic respiration by the symbionts.

### Carbon Fixation and Heterotrophy

All of the symbiont genomes encoded genes for the fixation of inorganic carbon. The γ*-proteobacterial* symbiont genomes each contained the genes for the Calvin-Benson-Bassham (CBB) cycle, including genes for a Form II RubisCO (*cbbM*). The *A*. ε symbiont genome had the genes for key enzymes involved in the reductive tricarboxylic acid (rTCA) cycle: ATP-citrate lyase (*aclAB*), 2-oxoglutarate:ferredoxin oxidoreductase (*oorABCD*), and fumarate reductase/succinate dehydrogenase (SDHAB), as well as all other genes necessary for rTCA except for the gene encoding phosphoenolpyruvate (PEP) synthetase (*ppsA*). The pathways found in each symbiont genome are typical of their taxonomic groups (i.e., *Campylobacteria* and γ*-proteobacteria*) (Hügler and Sievert, 2011). Further, the stable isotopic fingerprints of these pathways are reflected in the carbon stable isotopic composition of *Alviniconcha* species and *I. nautilei*, with *A. boucheti* (hosting the *A*. ε symbiont) having a much less negative ∂^13^C than the γ*-proteobacteria* hosting *Alviniconcha* species or *I. nautilei* (Beinart et al., 2012, 2015). Though the CBB cycle is more commonly found in aerobic organisms, while the rTCA cycle is more typical in microaerophilic or anaerobic organisms (Hügler and Sievert, 2011), oxygen concentrations around *Alviniconcha* species are not significantly different (Beinart et al., 2012) and *Alviniconcha* species are found at lower oxygen concentrations then *I. nautilei*, regardless of which symbiont they host (Podowski et al., 2009, 2010). Thus, an interaction between carbon fixation pathways and oxygen habitat is not likely to be important to habitat partitioning in these species.

In addition to autotrophy, all of the snail symbiont genomes indicated the capacity for heterotrophy. All of the symbiont genomes contained the genes for tripartite ATP-independent periplasmic (TRAP) dicarboxylate transporters, which would allow for the acquisition of four-carbon compounds like succinate and fumarate (Mulligan et al., 2011). In addition, both the Ifr1 and the *A*. γ-1 symbiont genomes contained the gene for an acetate permease (*actP*), which can transport both acetate and glycolate (Gimenez et al., 2003). The Ifr1 symbiont genome also contained two genes for cytoplasmic proteins involved in the sugar-transporting phosphotransferase system (PTS) (*ptsHI*). However, the lack of any genes for PTS permeases indicates that the PTS system may function in regulation in these bacteria, not in the import of carbon compounds (Barabote and Saier, 2005).

### Nitrogen Assimilation

The four symbiont genomes encoded genes for the assimilation of both nitrate and ammonium into biomass. All of the symbiont genomes encoded genes for an ammonium transporter and a nitrate/nitrate transporter, which suggests these bacteria could take up ammonium or nitrate from the environment (as well as take up any ammonium that is produced by the host). All of the symbiont genomes encoded genes for the reduction of nitrate in the periplasm (*napABGH*), and for the transport of the resulting nitrite into the cytoplasm (nitrate/nitrate transporter). Nap nitrate reductases are functionally diverse and have been implicated in both nitrate respiration and nitrate scavenging (Sparacino-Watkins et al., 2014). The symbiont genomes all contained cytoplasmic nitrite reductases that produce ammonia from nitrite for assimilation, though the genes were different: the γ*-proteobacterial* symbionts had the genes for a nitrite reductase (nit-6) that utilizes NAD(P)H as the reductant, while the *A*. ε symbiont contained a reductase that utilizes ferredoxin (*nirA*). All the symbionts had genes encoding a glutamine synthetase type I (*glnA*) for the conversion of ammonia to glutamine, and an NADPH-dependent glutamate synthase (*gltAB*) to convert glutamine to glutamate (though the *gltB* gene was not recovered in the Ifr1 symbiont genome). In addition, the Ifr1 symbiont genome contained a gene for a ferredoxin-dependent glutamate synthase (*glsF*).

### Amino Acid Synthesis and Transport

Though *Alviniconcha* and *I. nautilei* have gastrointestinal tracts, these systems are noticeably reduced and thought to be mostly non-functional (Beck, 1991; Waren and Bouchet, 1993). Thus, it is likely that all essential amino acids (i.e., amino acids not able to be synthesized by animals) must be provided by their symbionts, either through digestion of symbiont cells or via the excretion of amino acids by symbionts. All of the symbiont genomes encoded the complete or almost complete (i.e., missing 1 or 2 genes but encoding the gene for the final step) pathways to synthesize 15 amino acids, of which seven are considered essential to animals (Table 3). Additionally, all of the *Alviniconcha* symbionts contained the complete pathways to synthesize the two other essential amino acids, leucine and isoleucine (Table 3). Though the Ifr1 genome was incomplete for the synthesis of these two essential amino acids, it contained all genes in these pathways except for the final step (a branched-chain amino acid aminotransferase), which could indicate that the lack of this gene is due to the incompleteness of the Ifr1 genome assembly.

**TABLE 3:** Amino acid biosynthesis pathways in the four symbiont genomes.

**Biosynthesis of**	**Input substrate**	**A. ε**	***A*. γ-1**	***A*. γ-Lau**	**Ifr1**
Alanine	Valine	Inc	C	C	C
	Glutamate	Inc	Inc	Inc	Inc
	Cysteine	C	C	Inc	Inc
Arginine^∗^	Glutamate	C	C	C	C
Asparagine	Aspartate	C	C	C	C
Aspartate	Oxaloacetate	C	C	C	Inc
Cysteine	Homocysteine and serine	Inc	Inc	Inc	Inc
	Serine	C	Inc	Inc	Inc
Glutamate	Oxoglutarate	Inc	C	Inc	C
	Glutamine	C	C	C	C
Glutamine	L-glutamate	C	C	C	C
Glycine	Threonine	Inc	Inc	Inc	C
Histidine^∗^	PRPP	C	C	AC	C
Isoleucine^∗^	AcetylCoA and pyruvate	AC	AC	AC	Inc
	Threonine and pyruvate	AC	AC	AC	Inc
Leucine^∗^	Oxoisovalerate	C	C	C	Inc
Lysine^∗^	AcetylCoA	Inc	Inc	Inc	Inc
	Diaminoheptanedioate	C	C	C	C
Methionine^∗^	Homocysteine	C	C	C	C
Phenylalanine^∗^	Chorismate	C	C	C	C
Proline	Glutamate	C	C	C	C
Serine	Glycerate3P	C	C	C	C
	Glycine	C	C	C	C
Threonine^∗^	Homoserine	C	C	AC	AC
Tryptophan	Chorismate	C	C	C	C
Tyrosine	Chorismate	C	C	C	C
Valine^∗^	Oxoisovalerate	C	C	C	C

*^∗^Amino acid considered essential to animals. C, complete pathway; AC, almost complete pathway: pathway missing 1 or 2 genes but gene for final step is present; Inc, incomplete pathway.*

The *A*. γ-Lau and Ifr1 symbiont genomes both indicated the capacity for the uptake or excretion of amino acids. Though all of the symbiont genomes contained genes involved in dipeptide transport (*dppAC* was present in all *Alviniconcha* symbiont genomes and *dppA* only in the Ifr1 symbiont genome), they are missing the genes necessary for the complete dipeptide transport system (i.e., *dppBDFP*). The function of this partial dipeptide transport genes is therefore unclear. However, the *A*. γ-Lau symbiont genome contained genes for a branched-chain amino acid transporter, an L-amino acid ABC transporter (*aapJMQ*), and all genes for a cluster 5 ABC transporter that could transport peptides. The Ifr1 symbiont uniquely encoded an uncharacterized amino acid permease from the GabP family, as well as all the genes for an oligopeptide transport system (*oppABCDF*), which would allow it to take up peptides to use as amino acid sources. Since not all twenty amino acids were able to be synthesized by the symbionts, it is possible that they use these transporters to take up peptides or amino acids from their hosts or the environment. It is also possible that these transporters are being used by the symbionts for the export of peptides or amino acids to their hosts. However, the presence of symbiont-derived fatty acids in host tissue has been used as evidence to hypothesize that the γ*-proteobacterial* symbionts of *Alviniconcha* digest their symbionts, rather than rely on the translocation of substrates across the membrane to the host (Suzuki et al., 2005b).

### Motility and Adhesion

Though non-motile while intracellular, the symbionts of *Alviniconcha* spp. and *I. nautilei* are thought to be horizontally transmitted and, thus, have a free-living stage where motility could be useful for moving to optimal environmental habitats or for finding a host. Moreover, in pathogenic and symbiotic bacteria, motility machinery is often used for adhesion to or interaction with their hosts (reviewed in Chaban et al., 2015). The *A.* ε symbiont genome was the only symbiont genome that encoded genes for flagella, including genes for the flagellar hook capping protein (*flgD*), filament (*fliC*), hook-filament junction (*flgLK*), hook (*flgE*), rod (*flgBCGF*, *fliE*), ring (*flgHI*, *fliFGMN*), and motor (*motAB*). This genome also contained the paralyzed flagellar protein (*pflaA*) that is unique to *Campylobacterota* flagella (Gupta, 2006). The presence of the genes for flagella implies that the *A.* ε symbiont can be motile during its free-living state outside of the host or possibly during host association. However, no genes for the chemotactic signaling system that is present in motile pathogenic *Campylobacterota* were found in the *A.*ε symbiont genome. Flagella and chemotaxis are important in both the colonization and adhesion of pathogenic *Campylobacterota* (Lertsethtakarn et al., 2011), though flagellar motility is primarily important for colonization of hosts (Gu, 2017). Interestingly, flagellar genes are expressed in host-associated *A.* ε symbionts (Sanders et al., 2013), suggesting that the symbiont is using the flagella as a secretion system or for penetration into host tissue; such functions have been observed in intracellular pathogens (Chaban et al., 2015).

Both the *A*. γ-Lau and Ifr1 symbiont genomes encoded genes for type IV pili, which are involved in bacterial motility, adhesion, DNA uptake, and protein secretion (Grohmann et al., 2018). The *A*. γ-Lau symbiont genome encoded genes for type IV pili biogenesis (*pilMNOPY1Z*), secretin (*pilQ*), cassette (*pilS*), integral membrane protein (*pilC*), ATPases (*pilBTU*), and twitching motility proteins (*pilGHJT*). The Ifr1 symbiont shared all of these genes except *pilP*, *pilS*, and *pilY1*. Interestingly, the gene encoding pilin (*pilA*), the major structural component of the pilus, was missing from both genomes. Loss of this gene in pathogens impacts virulence (e.g., Essex-lopresti et al., 2005; Forslund et al., 2010), so it is not clear what the lack of this gene indicates for the functioning of the encoded type IV pili in these two symbionts.

### Secretion

The *A*. γ-1 and Ifr1 symbiont encoded genes for secretion systems (T4SSs), which are used to export DNA or proteins into cells and are common in intracellular bacteria (Alvarez-Martinez and Christie, 2009). The *A*. γ-1 symbiont genome encoded all necessary genes for a type I secretion system (*hlyBD*, *tolc*), which is used in the export of a variety of molecules including digestive enzymes, heme-binding proteins, and pore-forming exotoxins (Green and Mecsas, 2016). The *A*. γ-1 symbiont genome also contained genes for a T4SS IncF plasmid conjugative transfer pilus (*traABCDEFGHIKLNUVW*). However, it was missing genes involved in DNA processing (*traMY*) and surface exclusion (*traST*), which suggests that this pilus may not be used for conjugation but instead for protein secretion (Firth et al., 1996). The Ifr1 symbiont had five of the twelve genes that would be necessary for a VirB/VirD4 T4SS (*virB4*, *virB8*, *virB10-11*, and *virD4*), a secretion system that is used for the transport of DNA or effector proteins in pathogens (Alvarez-Martinez and Christie, 2009).

## Summary and Conclusion

Here, we compared the gene content of distantly related bacteria that are symbiotic with sympatric and closely related host species. The presented genomes presented herein are between 87-96% complete, making it likely that most genes and pathways were recovered in the current assemblies. Nonetheless, the absence of a gene or pathway from these assemblies must be interpreted with caution.

Comparison of the genomes of the symbionts of *Alviniconcha* species and *I. nautilei* demonstrated that they were highly similar in their gene content encoding for chemoautotrophic functions. All of the symbiont genomes contained genes for carbon fixation, oxidation of both sulfur and hydrogen, and the respiration of oxygen and nitrate. In most cases, the exact complement of genes found in these pathways was congruent with the metabolic pathways typically employed by other bacteria in their respective taxonomic groups. In addition, all four symbionts encoded genes for the uptake of organic carbon, indicating a capacity for heterotrophy, as well as the assimilation of nitrate and ammonia. The symbiont genomes did differ in gene content that may be related to interactions with their hosts, such as genes related to motility, adhesion, amino acid uptake or excretion, and secretion.

Altogether, our results suggest that, at the broadest level, differences in the presence or absence of the capacity for chemoautotrophic functions (e.g., genes for sulfur and/or hydrogen oxidation, as well as for aerobic and anaerobic respiration) do not of themselves explain the distribution of *Alviniconcha* and *I. nautilei* and their symbionts. This is important to consider when using genomic or metagenomic content as a proxy for function; the presence of nearly identical genes among different symbionts does not in and of itself imply comparable metabolic activity. It is likely that the particular homologs or pathways employed by these symbionts vary in their regulation and/or biochemical efficacies under different geochemical regimes, which could easily affect the distribution of their hosts into distinct habitats. For example, though all symbiont genomes contained sulfide:quinone reductase (SQR) genes, differences in the functioning of the types of SQR encoded in the *A*. ε symbiont compared to the γ*-proteobacterial* symbiont genomes could influence the optimal hydrogen sulfide concentration for the species hosting this symbiont. Though comparisons of the conditions around the different *Alviniconcha* species (Beinart et al., 2012), and physiological experiments testing differences between *Alviniconcha* and *I. nautilei* (Henry et al., 2008) do not suggest that there are interspecific differences in temperature or oxygen concentration tolerances, we cannot exclude the possibility that differences in host physiology or ecology are driving habitat partitioning in these species. Further work that assesses gene and protein expression, as well as directly compares the metabolism of these symbioses under common conditions, will provide insight into whether physiological differences among the host species and/or their symbionts is impacting their distribution.

## Data Availability

Raw sequence data can be found under the NCBI BioProject ID PRJNA523619, and NCBI BioSample IDs SAMN10985003 (*A. boucheti*), SAMN10985004 (*A. kojimai*), SAMN10985005 (*A. strummeri*), and SAMN10985044 (*I. nautilei*). GenBank SRA accession numbers for the sequence reads are SRR8632448 (*A. boucheti*), SRR8655138 (*A. kojimai*), SRR8799392 (*A. strummeri*), and SRR8799391 (*I. nautilei*). The assembled and annotated symbiont genomes are publicly available on the RAST server (http://rast.theseed.org/) using the guest login with IDs 6666666.293770 (*A*. γ-Lau), 6666666.293769 (*A*. γ-1), 6666666.293768 (*A.* ε), 6666666.293767 (Ifr1), and 6666666.296237 (Ifr2).

## Author Contributions

RB and PG designed the study. RB performed the laboratory work, assembly and binning of the *Ifremeria nautilei* symbiont genomes, and all other sequence analysis. CL and KK developed the new algorithm for separating host and symbiont sequence reads, and assembled the *Alviniconcha* symbiont genomes. FS sequenced *Ifremeria nautilei* symbiont genome. All authors contributed to the writing of the manuscript.

## Conflict of Interest Statement

The authors declare that the research was conducted in the absence of any commercial or financial relationships that could be construed as a potential conflict of interest.

## Footnotes

1 https://github.com/FelixKrueger/TrimGalore
