# Supplementary material for: The Bacterial Symbionts of Closely Related Hydrothermal Vent Snails With Distinct Geochemical Habitats Show Broad Similarity in Chemoautotrophic Gene Content
Source: Front Microbiol. 2019 Aug 14;10:1818. doi: 10.3389/fmicb.2019.01818 (PMC6702916; doi:10.3389/fmicb.2019.01818)
Supplement: Supplementary file 1 [file Table_1.DOCX]

**Table S1:** Summary of gene content for each symbiont genome.

| **Symbiont genome** | **Total CDS (unique annotations/hypothetical)** | **Repeat regions** | **tRNA genes (unique)** | **5S rRNA gene** | **16S rRNA gene** | **23S rRNA gene** |
| --- | --- | --- | --- | --- | --- | --- |
| *A*. ε | 2072 (1130/826) | 97 | 41 (31) | 0 | 1 | 2 |
| *A.* γ-1 | 4214 (1573/2279) | 223 | 39 (34) | 0 | 1 | 1 |
| *A.* γ-Lau | 2280 (1253/891) | 29 | 41 (39) | 1 | 1 | 1 |
| Ifr1 | 3615 (1555/1625) | 23 | 32 (30) | 1 | 1 | 1 |
