# Supplementary material for: The Bacterial Symbionts of Closely Related Hydrothermal Vent Snails With Distinct Geochemical Habitats Show Broad Similarity in Chemoautotrophic Gene Content
Source: Front Microbiol. 2019 Aug 14;10:1818. doi: 10.3389/fmicb.2019.01818 (PMC6702916; doi:10.3389/fmicb.2019.01818)
Supplement: Supplementary file 3 [file Table_3.DOCX]

**Table S3:** Abundance of shared core genes by SEED category, ordered from most to least abundant.

| **Category** | **Gene Count** |
| --- | --- |
| Uncategorized | 429 |
| Protein Metabolism | 116 |
| Amino Acids and Derivatives | 60 |
| Cofactors, Vitamins, Prosthetic Groups, Pigments | 51 |
| Respiration | 48 |
| DNA Metabolism | 39 |
| RNA Metabolism | 29 |
| Carbohydrates | 25 |
| Nitrogen Metabolism | 24 |
| Fatty Acids, Lipids, and Isoprenoids | 20 |
| Virulence, Disease and Defense | 18 |
| Membrane Transport | 17 |
| Stress Response | 14 |
| Nucleosides and Nucleotides | 13 |
| Sulfur Metabolism | 13 |
| Cell Wall and Capsule | 10 |
| Miscellaneous | 6 |
| Phosphorus Metabolism | 4 |
| Regulation and Cell signaling | 4 |
| Secondary Metabolism | 4 |
| Phages, Prophages, Transposable elements, Plasmids | 2 |
| Metabolism of Aromatic Compounds | 1 |
| Potassium metabolism | 1 |
